# Therapeutic Monitoring of Vancomycin and Factors Affecting Survival in ICU Patients with Infections

**DOI:** 10.3390/ph19081303

**Published:** 2026-08-18

**Authors:** Nadezhda G. Berdnikova, Dmitry V. Tsyganko, Vladislav D. Vasiukov, Vladimir S. Arnautov, Aleksei V. Trofimov, Valerii A. Menshov, Evgeniy S. Melnikov, Evgenia V. Shikh, Susanna S. Sologova, Elena A. Smolyarchuk, Natalia B. Lazareva

**Affiliations:** 1 Department of Clinical Pharmacology and Internal Diseases, Sechenov First Moscow State Medical University (Sechenov University), Moscow 119991, Russia; berdnikova_n_g_1@staff.sechenov.ru (N.G.B.); chih@mail.ru (E.V.S.); natalia.lazareva@gmail.com (N.B.L.); 2 Pulmonary Department, City Clinical Hospital Named After I.V. Davydovsky, Moscow 109240, Russia; tsygankodv@zdrav.mos.ru; 3 Department of Industrial Pharmacy, National Research Nuclear University “MEPhI”, Moscow 115409, Russia; vladislav3861@gmail.com (V.D.V.); riftquod@gmail.com (V.S.A.); 4 Laboratory for Radiobiology & Ecology, Emanuel Institute of Biochemical Physics, Russian Academy of Sciences, Moscow 119334, Russia; avt_2003@mail.ru (A.V.T.); vinoman66@mail.ru (V.A.M.); 5 Department of Pharmaceutical and Toxicological Chemistry, Sechenov First Moscow State Medical University (Sechenov University), Moscow 119991, Russia; melnikov_e_s@staff.sechenov.ru; 6 Department of Pharmacology, A.P. Nelyubin Institute of Pharmacy, Sechenov First Moscow State Medical University (Sechenov University), Moscow 119991, Russia; smolyarchuk_e_a@staff.sechenov.ru

**Keywords:** vancomycin, therapeutic drug monitoring, intensive care unit, pharmacokinetics, trough concentration, survival analysis, Cox regression, acute kidney injury, MRSA, nephrotoxicity

## Abstract

**Background/Objectives:** Vancomycin remains a cornerstone antibiotic for Gram-positive infections, but its narrow therapeutic window and marked pharmacokinetic variability in critical patients complicate dosing. This study aimed to analyze survival and identify factors associated with mortality in intensive care unit (ICU) patients receiving vancomycin under therapeutic drug monitoring (TDM). **Methods:** A single-center, retrospective cohort study was conducted at City Clinical Hospital named after I.V. Davydovsky (Moscow, Russia) between December 2021 and January 2026 (IRB protocol VANCO-2021). A total of 190 adult patients from surgical, therapeutic, and cardiac ICUs receiving vancomycin for at least 3–5 days were analyzed; trough concentrations were determined by HPLC-MS/MS. Cox proportional hazards, spline Cox, and segmented (piecewise) Cox models were applied to identify mortality predictors, with bootstrap subsampling assessing threshold robustness. **Results:** Three independent predictors of mortality were identified: ln(minimum vancomycin concentration) (HR = 1.644, *p* = 0.028), age (HR = 1.019, *p* = 0.037), and serum creatinine (HR = 1.004, *p* < 0.001). A data-driven threshold trough concentration (Ctrough) of 25.5 µg/mL was identified, above which mortality increased substantially (63.0% vs. 26.4%; *p* < 0.05). The adjusted hazard ratio for Ctrough ≥ 25.5 µg/mL was approximately 1.88 (*p* = 0.054); bootstrap subsampling yielded an uncertainty interval of 23.2–33.6 µg/mL. **Conclusions:** Elevated minimum vancomycin concentrations exceeding 25.5 µg/mL are independently and statistically associated with increased mortality in ICU patients, likely reflecting altered drug exposure and illness severity rather than a direct causal effect. Systematic TDM is essential for maintaining vancomycin within the therapeutic range.

## 1. Introduction

Vancomycin remains a first-line agent against Gram-positive infections owing to its proven efficacy and broad clinical experience. However, its narrow therapeutic window makes optimal dosing challenging in clinical practice. Trough concentrations below 10–20 µg/mL are associated with clinical failure and the emergence of antimicrobial resistance [1,2]. Conversely, overexposure substantially increases the risk of nephrotoxicity and ototoxicity. This risk is particularly pronounced in patients with impaired renal function and a prolonged elimination half-life. Consequently, the pharmacokinetic characteristics of vancomycin continue to be actively investigated, with a focus on patients presenting diverse anthropometric features, clinical conditions, and comorbidities.

In critical patients in intensive care units, marked variability is observed in the concentrations achieved when the drug is administered according to standard protocols, which frequently has a decisive impact on disease outcome. Such patients may exhibit a very large volume of distribution (Vd) owing to endothelial dysfunction leading to capillary leak and to massive fluid resuscitation, as well as supranormal drug clearance, both of which result in a substantial decline in concentration and, accordingly, in reduced treatment efficacy. Conversely, low values of the glomerular filtration rate necessitate reduced dosages or extended dosing intervals [3,4,5].

Vancomycin therapy is guided by therapeutic drug monitoring (TDM), which, in serious infections caused by resistant *Enterococcus* spp., *Staphylococcus* spp., and *Streptococcus* spp., has historically been based on a target trough plasma concentration of 15–20 µg/mL, used as a surrogate for an area-under-the-curve (AUC) to minimum inhibitory concentration (MIC) ratio of ≥400 mg·h/L, assuming an MIC of 1 mg/L. In the 2020 consensus guidelines on vancomycin, this recommendation was revised to a target AUC:MIC ratio of 400–600 mg·h/L. Owing to insufficient data supporting the safety of maintaining trough concentrations of 15–20 µg/mL, earlier Japanese guidelines proposed initiating therapy at a dose sufficient to achieve trough concentrations of 10–15 µg/mL even in patients with complicated infections [6]. According to the guidelines of the Infectious Diseases Society of America, a loading dose of 25–30 mg/kg (actual body weight) and a prolonged infusion of up to 2 h may be considered in critical patients with suspected MRSA infection (e.g., sepsis, meningitis, pneumonia, or infective endocarditis) [7].

To optimize the dosing regimen, freely available Bayesian pharmacokinetic calculators have been developed [8,9], which allow individualization of vancomycin dosing on the basis of patient anthropometric and laboratory parameters. In routine clinical practice, however, situations are encountered in which such calculators are unable to adequately recommend an optimal regimen—notably in the settings of renal replacement therapy, unstable renal function, a vancomycin MIC greater than 2 µg/mL, or an age below 18 years [10,11].

It is well established that sepsis carries a risk of acute kidney injury (AKI), a prognostically unfavorable complication in critical patients. A European multicenter study reported that AKI developed in 51% of septic ICU patients, with a mortality of 41% among those with AKI; patients with septic AKI had more severe disease, higher mortality, and a greater need for dialysis than patients with non-septic AKI [12,13]. Failure of renal recovery within the first 7 days after the diagnosis of AKI has been identified as an independent predictor of 90-day mortality [14].

Given the substantial impact of AKI on clinical outcomes and the well-recognized nephrotoxic effects of vancomycin, together with its pharmacokinetic unpredictability, TDM of this antibiotic is essential in critical patients.

We hypothesized that elevated vancomycin trough concentrations, rather than dosing regimen alone, independently predict mortality in critical patients, and that this relationship may be non-linear with an identifiable threshold.

The present study had the following objectives: (1) to analyze survival in ICU patients with infectious diseases receiving vancomycin under TDM; (2) to identify the most significant factors affecting patient survival; and (3) to determine the pharmacokinetic parameters potentially influencing survival.

## 2. Results

Building on the study rationale outlined above, we first characterized the baseline cohort before proceeding to survival analysis.

190 patients from surgical, therapeutic, and cardiac ICUs were included in the study, with subsequent follow-up of patients who were transferred to general wards. The actual weight-based vancomycin dose ranged from 20 to 45 mg/kg/day; the most frequently administered dose was 1.5–2 g/day by intravenous infusion. The duration of antibacterial therapy was 10.3 ± 3.5 days.

Table 1 presents the baseline demographic, anthropometric, laboratory, and pharmacokinetic characteristics of the study population. The cohort was characterized by pronounced interindividual variability in renal function and vancomycin exposure, including a wide range of serum creatinine values and plasma drug concentrations.

Table 2, Table 3 and Table 4 show the distribution of patients by site of infection, identified pathogens, and comorbidities. The most frequent infections were skin and soft tissue infections, pneumonia, and sepsis, whereas the predominant pathogens were *Staphylococcus aureus*, *Klebsiella pneumoniae*, and *Acinetobacter baumannii*. The study population was also characterized by a high comorbidity burden.

### 2.1. Multivariable Survival Analysis: Cox Proportional Hazards Model

Table 5 presents the results of the Cox regression assessing factors associated with the risk of death. The final model included the natural logarithm of the minimum vancomycin concentration, patient age, and serum creatinine level. All included variables were statistically significant predictors of mortality. According to the model, an increase in the natural logarithm of vancomycin Ctrough was associated with an increased risk of death (HR = 1.644; *p* = 0.028). Age was also a statistically significant predictor of an unfavorable outcome (HR = 1.019; *p* = 0.037), while serum creatinine demonstrated the strongest statistical significance (HR = 1.004; *p* < 0.001).

### 2.2. Threshold Analysis: Ctrough Cut-Off of 25.5 µg/mL

**Figure 1:**
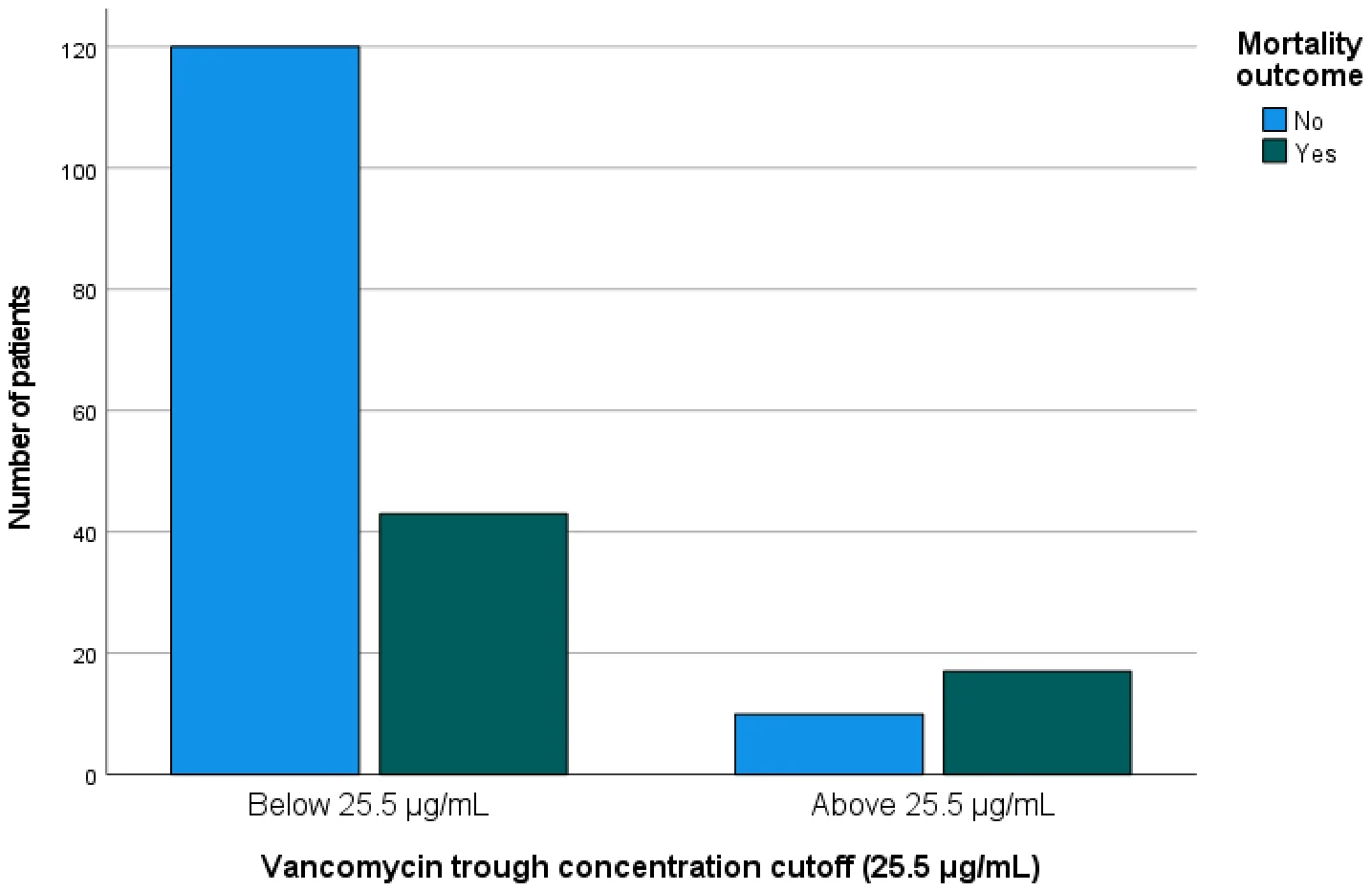
Mortality distribution by cut-off group (<25.5 vs. ≥25.5 µg/mL).

Table 6 illustrates the relationship between the vancomycin Ctrough and mortality. The analysis identified a potential threshold region at approximately 25.5 µg/mL. When patients were categorized according to this cut-off, death occurred in 26.4% of patients with a Ctrough below 25.5 µg/mL and in 63.0% of patients with a Ctrough above this value. The differences between groups were statistically significant by the Pearson χ^2^ test and Fisher’s exact test. The distribution of fatal outcomes relative to the 25.5 µg/mL threshold is shown in Figure 1.

**Figure 2:**
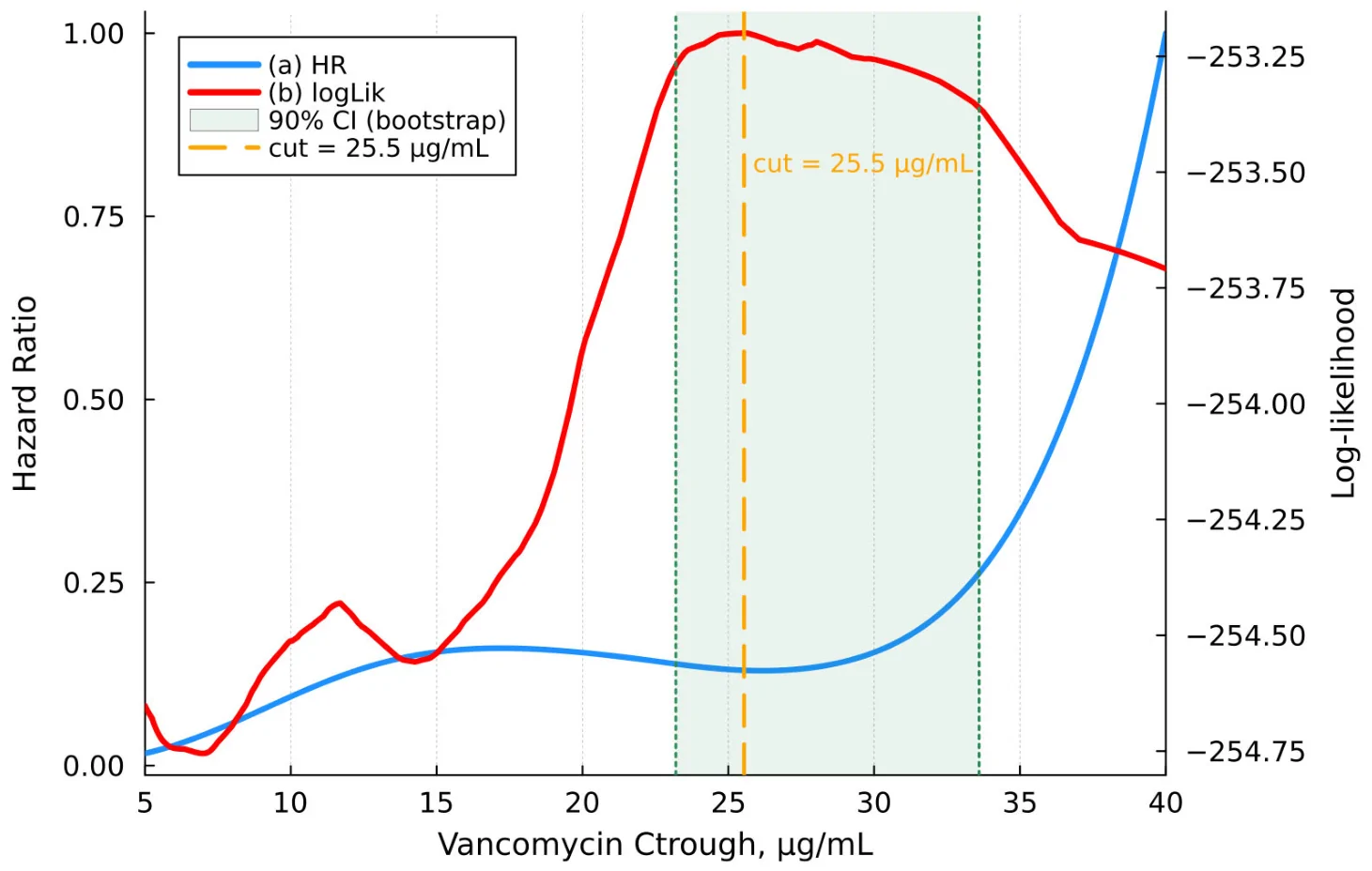
(**a**) Blue line—dependence of the hazard ratio on ln(Ctrough), estimated by spline Cox; (**b**) red line—dependence of the log-likelihood function on the selected cut-off, with the maximum and 90% CI indicated.

Figure 2a shows the dependence of the hazard ratio (HR) on the vancomycin trough concentration (Ctrough), estimated using a Cox proportional hazards model with spline approximation (spline Cox; blue curve). Figure 2b shows the dependence of the partial log-likelihood function on the analyzed threshold concentration, obtained within a segmented (piecewise) Cox model (red curve). The solid vertical line corresponds to the optimal threshold value of Ctrough = 25.5 µg/mL, at which the likelihood function reaches its maximum; the dashed vertical lines (green) denote the boundaries of the 90% confidence interval of this estimate.

### 2.3. Adjusted Model with a Binary Concentration Predictor

Table 7 presents the Cox regression coefficients with Ctrough included as a categorical predictor (<25.5 µg/mL vs. ≥25.5 µg/mL), adjusted for age and serum creatinine. Patients with a Ctrough ≥ 25.5 µg/mL had a higher risk of death; the adjusted hazard ratio was approximately 1.88 (*p* = 0.054).

**Figure 3:**
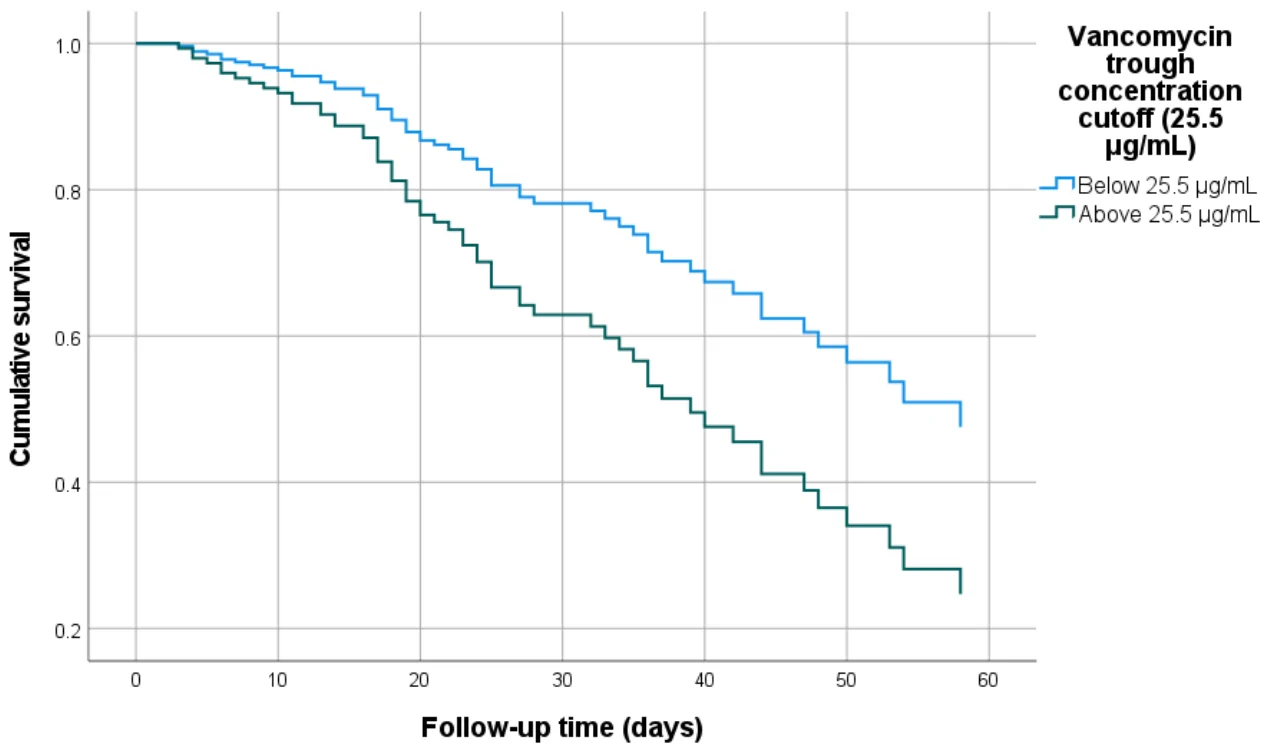
Cox survival functions for the groups with Ctrough < 25.5 and Ctrough ≥ 25.5 µg/mL (adjusted for age and serum creatinine).

Figure 3 shows the survival functions derived from the Cox proportional hazards model, adjusted for age and serum creatinine, for the two patient groups stratified by the 25.5 µg/mL Ctrough threshold. Patients with a Ctrough ≥ 25.5 µg/mL exhibited lower estimated survival throughout the entire follow-up period (HR ≈ 1.88; *p* = 0.054).

## 3. Discussion

These findings should be interpreted in the context of existing evidence on vancomycin exposure–toxicity relationships. Although vancomycin was administered in accordance with current recommendations, more than 50% of hospitalized patients failed to maintain concentrations within the target range (10–20 µg/mL) after steady state was reached. This indicates that determining the vancomycin dose solely based on age, weight, renal function, infection severity, and pathogen susceptibility does not ensure satisfactory serum concentrations in most patients. Treatment failures have been reported in patients with MRSA infections despite in vitro susceptibility. To achieve target vancomycin concentrations within the 10–20 µg/mL range recommended by several IDSA-endorsed clinical practice guidelines, higher doses are administered; however, increased rates of nephrotoxicity have been associated with aggressive vancomycin use and are aggravated by concomitant nephrotoxic agents, renal insufficiency, and/or hemodynamic alterations [15,16,17]. These observations are also reflected in other meta-analyses (<15 vs. ≥15 mg/L), which showed that nephrotoxicity occurred on average 4–17 days after the initiation of vancomycin therapy, with an incidence ranging from 5% to 43%; this wide range reflects population characteristics, in particular ICU stay, concomitant use of other nephrotoxic drugs, and additional predisposing factors (obesity, comorbidity, renal insufficiency). The management of vancomycin dosing extends beyond the assessment of efficacy and safety, with growing interest focused on the prediction of survival. The relationship between vancomycin-induced nephrotoxicity and mortality has not been firmly established; nevertheless, a correlation between vancomycin-induced nephrotoxicity and mortality has been observed in patients with severe Gram-positive infections. A retrospective analysis of 337 patients with MRSA bacteremia treated with vancomycin identified nephrotoxicity as an independent risk factor for in-hospital mortality, unrelated to the vancomycin dose [18], which is consistent with the findings of the present study. Similar associations between antibiotic exposure and mortality have been reported for other agents used in critical patients with resistant Gram-negative infections, underscoring that this relationship is not unique to vancomycin [19,20]. In the present study, a preliminary analysis of factors potentially associated with mortality was performed, incorporating demographic, laboratory, clinical, and pharmacokinetic variables. Particular attention was paid to the minimum vancomycin concentration, as this parameter reflects residual drug exposure and may be related both to treatment efficacy and to the risk of toxicity. A baseline Cox proportional hazards model was constructed in which the natural logarithm of the minimum vancomycin concentration, patient age, and serum creatinine were included as independent variables in the final multivariable model; the minimum vancomycin concentration was thus associated with mortality independently of patient age and serum creatinine. It should be emphasized that most previous studies, including the one cited above, have linked elevated vancomycin trough concentrations (generally >20 µg/mL) to nephrotoxicity as the primary adverse outcome. The present study addresses a distinct endpoint—all-cause mortality—and, rather than applying a pre-specified concentration cut-off, derives the threshold empirically using spline Cox and segmented (piecewise) Cox modelling, with its robustness subsequently assessed by bootstrap subsampling (uncertainty interval 23.2–33.6 µg/mL, see Section 2.2). We consider this combination—a mortality-specific endpoint, a data-driven rather than pre-specified threshold, and a combined surgical, therapeutic, and cardiac ICU cohort—to constitute the principal element of novelty of this work relative to previously published trough–nephrotoxicity associations.

At the same time, the association identified between elevated Ctrough and mortality should be interpreted as statistical rather than causal. Elevated trough concentrations may reflect reduced drug clearance secondary to renal dysfunction, greater underlying illness severity, or altered volume of distribution, rather than exerting a direct toxic effect leading to death; accordingly, vancomycin Ctrough in this cohort should be regarded as a marker of drug exposure and physiological derangement rather than as an independent causal determinant of mortality. This interpretation is further supported by the fact that critical patients frequently exhibit capillary leak and receive massive fluid resuscitation, both of which increase the volume of distribution and can lower measured concentrations independent of dosing, while simultaneously serving as markers of illness severity [3].

It is important to note that subtherapeutic vancomycin concentrations carry a distinct and clinically significant risk—namely, treatment failure and the potential selection of resistant strains, particularly in MRSA infections [1,2]. This risk represents a mechanistically different pathway to poor outcome compared with overexposure-related toxicity. The present threshold analysis was specifically designed to identify an upper concentration boundary associated with excess mortality risk and was not intended to characterize the lower boundary of therapeutic failure. Future studies employing a U-shaped or J-shaped modeling approach across the full concentration range would help characterize both the sub- and supratherapeutic risk zones simultaneously.

In parallel, model-informed precision dosing (MIPD) approaches, such as those recommended by the Japanese Society of Chemotherapy [6], provide valuable tools for individualizing vancomycin dosing where Bayesian estimation software is available. The present findings are complementary to this approach: they provide observational evidence on the concentration–mortality relationship in a real-world ICU setting without routine access to MIPD infrastructure, and may therefore inform trough-based monitoring strategies in similarly resourced institutions.

When a segmented/piecewise Cox model was fitted, it was hypothesized that the relationship between the minimum vancomycin concentration and the risk of death might change beyond a certain threshold. To this end, a hinge component was included in the model, allowing the change in the slope of the relationship beyond the putative breakpoint to be estimated. The optimal breakpoint was determined by scanning the range of possible concentration values and selecting the model with the best partial log-likelihood. A Cox model with the concentration entered as a binary factor at the 25.5 µg/mL cut-off was then constructed. In the univariable model, this cut-off was statistically significantly associated with mortality; after adjustment for age and serum creatinine, the association retained clinical relevance but was of borderline statistical significance. Bootstrap subsampling showed that the identified cut-off point exhibited a degree of variability and should not be regarded as a rigid universal clinical threshold; the resulting uncertainty interval was approximately 23.2–33.6 µg/mL. Therefore, the value of 25.5 µg/mL should be interpreted as a statistically derived threshold region in this cohort, beyond which a more pronounced increase in the risk of death is observed.

It is worth noting that this point estimate is conservative and was identified based on the principle of selecting the model with a higher maximum log-likelihood value; statistical significance was not considered as the primary criterion for model selection. The identified threshold (25.5 µg/mL) was not regarded by us as an independent binary risk factor that must demonstrate statistical significance after variable binarization; rather, this value was obtained by searching for the point at which the nature of the relationship between the logarithm of the minimum vancomycin concentration and the risk of death changes within a segmented Cox model.

Overall, the study was performed in several sequential analytical stages: first, the structure of the cohort was described and a primary analysis of factors associated with mortality was conducted; a multivariable Cox model including ln(Ctrough), age, and serum creatinine was then constructed; subsequently, the functional form of the relationship between vancomycin concentration and the risk of death was examined using spline Cox and segmented Cox models; finally, the robustness of the identified threshold region was assessed by bootstrap subsampling.

This approach allowed not only the establishment of an independent association between an elevated minimum vancomycin concentration and mortality but also the characterization of the potentially nonlinear, threshold-dependent nature of this relationship.

### Study Limitations

This study has several limitations that should be acknowledged when interpreting the results. First, its retrospective, single-center design limits causal inference and generalizability to other institutions and populations; residual confounding by underlying disease severity cannot be excluded. Second, vancomycin monitoring was based on trough concentrations rather than Bayesian estimation of the AUC/MIC ratio. This reflects the standard of care at our institution during the study period (December 2021–January 2026), when Bayesian dosing software was not part of routine clinical practice. Although plasma vancomycin concentrations were sampled at three time points (pre-dose, 1 h, and 7 h after the end of infusion) for a subset of patients, these samples were obtained as part of routine trough-based TDM and a single pharmacokinetic profiling procedure on days 3–4 of therapy, rather than a structured protocol designed for population-pharmacokinetic AUC estimation. In the absence of a population pharmacokinetic model validated for our patient population, sampling scheme, and analytical method (HPLC-MS/MS), we did not perform a Bayesian AUC:MIC estimation; instead, we analyzed the trough concentration (Ctrough), which was the parameter that actually guided dose-adjustment decisions in clinical practice during the study period. We recognize that current consensus guidelines recommend AUC-guided monitoring where feasible, and consider a prospective, Bayesian AUC-guided analysis of this cohort—ideally using a locally validated population PK model—a priority direction for future work by our group, particularly in view of the 2020 consensus shift toward AUC:MIC-based dosing targets. Third, standardized ICU severity scores (SOFA, APACHE II), documented bacteremia status, and receipt of renal replacement therapy were not systematically recorded in the source dataset and could therefore not be incorporated into the multivariable model, despite their recognized prognostic relevance in critical patients. Fourth, the reported minimum inhibitory concentration (MIC) was not systematically standardized across all pathogens, which may have further affected the precision of AUC/MIC-related interpretations. Fifth, although individual comorbidities were recorded (Table 4), a composite comorbidity index (e.g., the Charlson Comorbidity Index) was not calculated. Finally, given the moderate sample size, machine learning-based survival modeling (e.g., decision trees, artificial neural networks) was not employed, as such methods typically require substantially larger event counts for stable and generalizable performance and carry a higher risk of overfitting in a dataset of this scale. These unmeasured factors and methodological constraints may act as residual confounders in the observed association between vancomycin trough concentration and mortality and should be considered when interpreting the proposed 25.5 µg/mL threshold, which we regard as a statistically derived region of increased risk in this cohort rather than a universally applicable clinical cut-off.

Future prospective, multicenter studies incorporating SOFA/APACHE II scores, bacteremia status, renal replacement therapy, AUC-based exposure metrics (ideally via Bayesian estimation), and a composite comorbidity index are warranted to validate and extend these findings.

## 4. Materials and Methods

### 4.1. Study Design and Patient Inclusion

This was a single-center, retrospective observational cohort study, reported in accordance with the STROBE guidelines for observational studies.

A retrospective cohort study was conducted at City Clinical Hospital named after I.V. Davydovsky between December 2021 and January 2026. The medical records of adult patients who had received vancomycin for at least 3–5 days were analyzed. Vancomycin was prescribed for the treatment of resistant Gram-positive infections caused by *Enterococcus* spp., *Staphylococcus* spp., and *Streptococcus* spp., according to microbiological data and/or clinical guidelines. The study enrolled patients from the surgical, therapeutic, and cardiac ICUs with documented infectious conditions, with subsequent follow-up of patients transferred to general wards. For each case, the following data were available: age, sex, actual body weight, diagnosis, type of infection and its microbiological characteristics, ICU/general-ward status, vancomycin dosing (single and daily dose), blood vancomycin concentration, comorbidity profile, laboratory results, and vital status (alive/dead). Inclusion criteria were: age ≥ 18 years; admission to a surgical, therapeutic, or cardiac ICU; a documented infection caused or suspected to be caused by *Enterococcus* spp., *Staphylococcus* spp., or *Streptococcus* spp.; and vancomycin therapy for at least 3–5 days with at least one measured trough concentration. Exclusion criteria were: age < 18 years; vancomycin therapy shorter than 3 days; chronic renal replacement therapy (hemodialysis or peritoneal dialysis) at admission; pregnancy; and incomplete medical records precluding determination of vital status or Ctrough. Of the patients screened during the study period, those meeting the exclusion criteria were not included in the final analytic cohort of 190 patients.

### 4.2. Dosing Regimen and Therapeutic Drug Monitoring

Vancomycin was prescribed in accordance with the summary of product characteristics at 15–20 mg/kg of body weight (with a maximum initial daily dose not exceeding 2 g), taking renal function into account on the basis of creatinine clearance estimated by the Cockcroft–Gault formula. Vancomycin was administered as a 60 min intravenous infusion every 8–12 h. The dosing regimen was adjusted after 24–48–72 h according to the glomerular filtration rate. Blood samples for the determination of the vancomycin trough concentration (Ctrough) were obtained within 30 min before the fifth dose. For the pharmacokinetic profile, blood was sampled 0.5 h before infusion, 1 h after, and 7 h after the end of infusion. Vancomycin concentrations were determined by high-performance liquid chromatography–tandem mass spectrometry (HPLC-MS/MS) in plasma on days 3–4 of therapy. Renal function was monitored continuously before and during vancomycin therapy. When the vancomycin Ctrough exceeded 25 mg/L, the dose was adjusted according to the patient’s condition, or the drug was switched to an alternative antibacterial agent. Nephrotoxicity was defined as an increase in serum creatinine (SCr) > 0.5 mg/dL (44.2 µmol/L) or an increase in SCr > 50% above baseline over at least 2 days [21].

### 4.3. Bioanalytical Method Validation

Vancomycin concentrations in patient plasma during TDM were quantified using reversed-phase high-performance liquid chromatography with tandem mass spectrometric detection (HPLC-MS/MS; Nexera liquid chromatography system coupled with an LCMS-8040 triple-quadrupole mass spectrometer, Shimadzu, Tokyo, Japan). Eremomycin was used as the internal standard to ensure the accuracy and precision of vancomycin quantification.

Chromatographic separation was performed on a Jupiter C18 column (50 × 4.6 mm, 5 µm, 300 Å; Phenomenex, Torrance, CA, USA) fitted with a universal C18 guard column (4 × 3.0 mm; Phenomenex, Torrance, CA, USA) at a column thermostat temperature of 40 °C. The mobile phase consisted of eluent A (0.1% *v*/*v* formic acid in deionized water) and eluent B (0.1% *v*/*v* formic acid in acetonitrile), delivered in gradient elution mode at a flow rate of 1.0 mL/min (Table 8). The injection volume was 3 µL. Ionization was performed by electrospray in positive-ion mode at an applied spray voltage of 5.0 kV; detection parameters for multiple reaction monitoring (MRM) were selected experimentally (Table 9).

Plasma samples were prepared by protein precipitation using an experimentally optimized precipitating agent—a 50% solution of trifluoroacetic acid (99%, Acros Organics, Geel, Belgium) in water. To 400 µL of plasma, 10 µL of internal standard solution (eremomycin, 800 µg/mL) was added, and the mixture was vortex-mixed for 15 s. Subsequently, 200 µL of the precipitating solution was added, the sample was vortexed for 1 min, and centrifuged for 15 min at 15,000 rpm. The supernatant was transferred to autosampler vials for chromatographic analysis. The confirmed analytical range of the method was 1–100 µg/mL.

**Figure 4:**
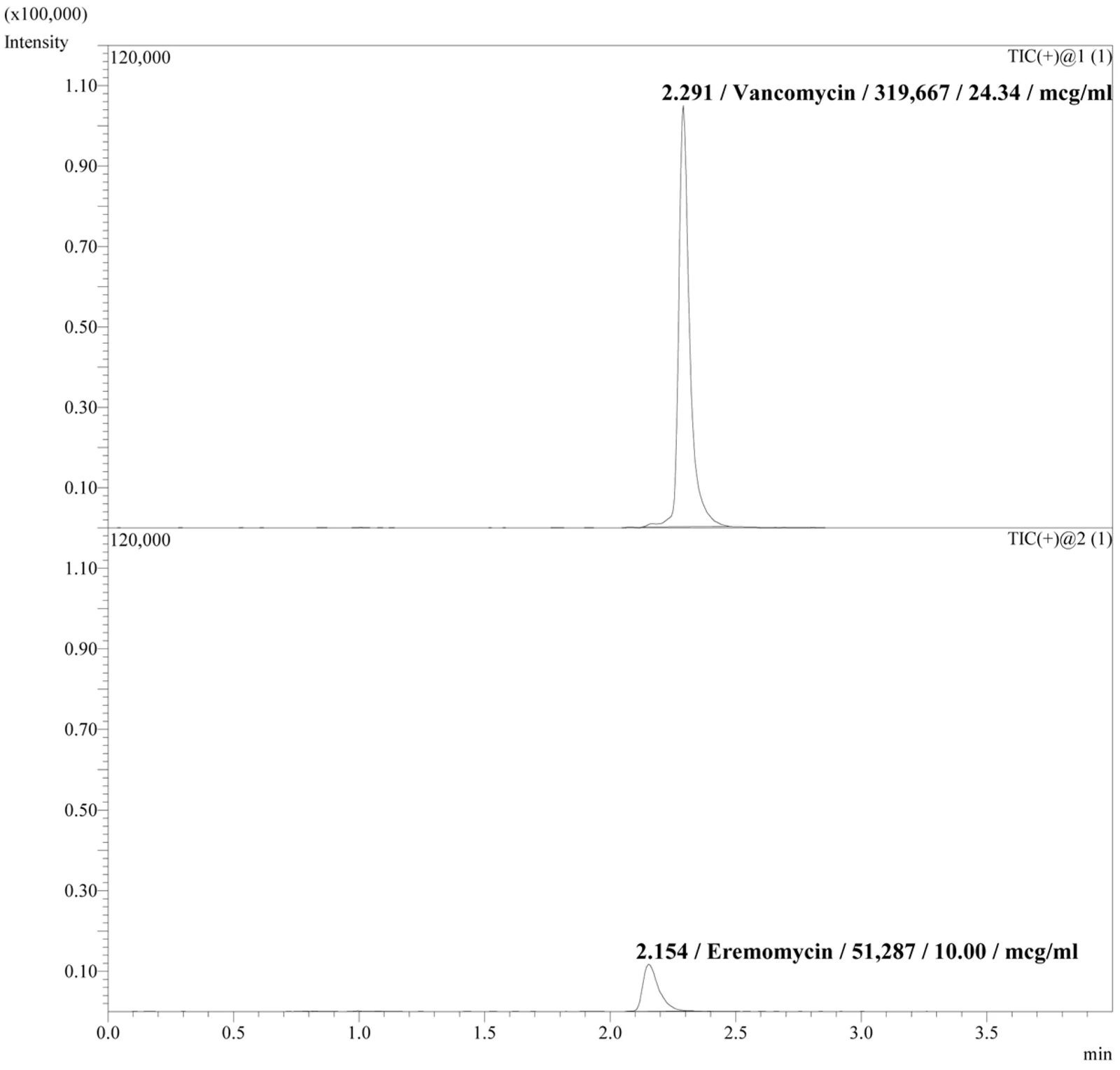
Representative multiple reaction monitoring (MRM) chromatogram (LabSolutions, Shimadzu) of a patient plasma sample, showing the vancomycin peak (retention time 2.291 min; 24.34 µg/mL) and the eremomycin internal standard peak (retention time 2.154 min; 10.00 µg/mL). Peak identity, retention time, and calculated concentration are annotated for each analyte.

Representative chromatograms illustrating the separation of vancomycin and eremomycin are presented in Figure 4. Eremomycin was chosen as the internal standard (IS) due to its structural similarity to vancomycin (both being glycopeptide antibiotics), ensuring comparable chromatographic retention, extraction recovery during protein precipitation, and ionization efficiency in positive electrospray ionization (ESI+) MRM mode. Eremomycin is not an approved pharmaceutical agent, precluding any therapeutic presence in patient plasma. Furthermore, because vancomycin and eremomycin are biosynthesized by distinct *Amycolatopsis* strains, eremomycin is not present as a related impurity in vancomycin drug formulations.

While stable isotope-labeled (SIL) standards (e.g., vancomycin-d12 or ^13^C-vancomycin) represent the benchmark for compensating matrix effects in LC-MS/MS bioanalysis, commercial availability and logistics constrained their immediate implementation. Consequently, a panel of structurally related glycopeptides (telavancin, oritavancin, ramoplanin, and eremomycin) was evaluated. Eremomycin demonstrated the closest retention behavior to vancomycin and was reliably accessible for routine clinical application. The working IS concentration was fixed at 10 µg/mL in plasma. While internal standards are conventionally added at concentrations yielding a response comparable to the mid-to-upper calibration range (e.g., equivalent to 30–50% of the ULOQ, corresponding to 30–50 µg/mL vancomycin), a lower concentration was deliberately selected to minimize potential carryover and conserve reference standard stocks. At 10 µg/mL, eremomycin yielded a signal equivalent to ~5 µg/mL vancomycin—aligning with typical clinical trough levels—while maintaining excellent analytical performance (mean signal-to-noise ratio ~200; intra-batch peak-area RSD ≤ 3%).

The method was developed and used as an in-house assay for routine clinical therapeutic drug monitoring (TDM) of vancomycin, guided by the U.S. Food and Drug Administration bioanalytical method validation guidance [22] and, more recently, by the internationally harmonized ICH M10 Guideline on Bioanalytical Method Validation and Study Sample Analysis [23]. Given the specific nature of therapeutic drug monitoring (TDM), where plasma samples are processed immediately upon receipt to provide rapid clinical feedback, long-term frozen sample storage and incurred sample reanalysis (ISR) were not applicable to the routine TDM workflow. However, to support short-term clinical logistics and potential re-analyses, short-term stability under relevant operational conditions was fully established.

*Selectivity, Matrix Effect and Recovery*

Method selectivity was demonstrated using six independent lots of normal blank human plasma, alongside one hemolyzed and one hyperlipidemic plasma lot. No interfering peaks at the retention times of vancomycin or eremomycin were observed in any matrix blank. Matrix factor (MF) and extraction recovery were evaluated across eight independent plasma sources at low (3.00 µg/mL) and high (75.00 µg/mL) quality control (QC) levels. The IS-normalized matrix factor was 0.93 (RSD 1.84%) for low QC and 0.92 (RSD 5.49%) for high QC, confirming that matrix-induced ionization suppression or enhancement was effectively compensated by eremomycin. The mean extraction recoveries were consistently high and reproducible (Table 10).

*Calibration Curve, Linearity, and Carryover*

Calibration curves were constructed over the concentration range of 1.00 to 100.00 µg/mL (1.00, 2.00, 3.00, 5.00, 10.00, 15.00, 20.00, 50.00, and 100.00 µg/mL) using 1/x^2^ weighted linear regression. The method demonstrated excellent linearity across the range, with back-calculated concentrations of calibration standards deviating by less than ±8.3% from nominal values (within the ±15% acceptance criteria, and ±20% at LLOQ). No carryover was detected in blank plasma samples injected immediately after the highest calibration standard (100.00 µg/mL).

*Accuracy and Precision*

Within-run (*n* = 5) and between-run (*n* = 15, across three analytical batches) accuracy and precision were evaluated at four QC levels: LLOQ (1.00 µg/mL), Low (3.00 µg/mL), Medium (30.00 µg/mL), and High (75.00 µg/mL). Across all evaluated levels, accuracy ranged from 88.11% to 103.76%, while precision (RSD) did not exceed 9.52% at LLOQ and remained below 5.1% for higher QCs (Table 11), fully satisfying FDA acceptance criteria.

*Stability*

Stability studies evaluated vancomycin integrity across pre-analytical, analytical, and storage steps (*n* = 3 per QC level). Vancomycin was found to be stable in plasma under bench-top conditions (room temperature, 6 h), post-preparation in the autosampler (+4 °C, 24 h), after three freeze–thaw cycles, and during frozen storage (−20 °C, 7 days). Stock and working solutions demonstrated stability for up to 193 days at −20 °C (Table 12).

*Dilution Integrity*

To enable the accurate quantification of clinical samples exceeding the upper limit of quantification (ULOQ > 100.00 µg/mL), dilution integrity was evaluated during partial method validation. Human plasma samples spiked with vancomycin at a concentration of 400.00 µg/mL (4× ULOQ) were diluted 4-fold with blank human plasma prior to extraction (*n* = 5). The mean back-calculated concentration after correcting for the dilution factor demonstrated an accuracy of 94.32% with a precision (RSD) of 4.25%, which fully complied with the acceptance criteria (accuracy within ±15% and RSD 15%). These data confirm that clinical samples with concentrations up to 400.00 µg/mL can be reliably quantified following 4-fold dilution with blank matrix without compromising method accuracy or precision.

*Routine Quality Assurance*

Each routine analytical run comprises a blank sample, a zero sample (matrix spiked with internal standard), a 9-point calibration curve, and duplicate QC samples at three concentration levels (low, medium, and high). Internal standard response stability and peak area drift are systematically monitored across the sequence to verify sample preparation consistency and instrument performance.

If an analytical run fails to meet predefined acceptance criteria, or if ambiguous analytical results occur (e.g., significant IS response deviation or unexpected concentration values), the sample is re-extracted and re-analyzed by an independent analyst.

### 4.4. Statistical Analysis

Statistical analysis was performed by constructing contingency tables, Cox proportional hazards regression models, spline Cox models, and segmented (piecewise) Cox models, with bootstrap subsampling used to assess the robustness of the identified threshold region. Complete-case analysis was used throughout: patients with missing values for any variable included in a given model (Ctrough, age, or serum creatinine) were excluded from that specific analysis, and no imputation was performed. The proportion of missing data for each variable in the final analytic cohort was below 5%. Cox proportional hazards regression, together with spline Cox and segmented (piecewise) Cox modeling, was selected over machine learning approaches (e.g., decision trees, artificial neural networks) given the sample size of the cohort (*n* = 190, 60 events). Regression-based survival models allow formal hypothesis testing, interpretable hazard ratios, and principled threshold estimation with bootstrap-derived confidence intervals, which are less readily achieved with black-box ML methods in datasets of this scale, where overfitting risk is substantial. Within this regression-based framework, spline Cox and segmented (piecewise) Cox models were chosen over restricted cubic splines with pre-fixed knots because the location of a possible breakpoint in the Ctrough–mortality relationship was not known a priori. A data-driven scan of candidate cut-offs, selecting the model with the highest partial log-likelihood, was therefore considered more appropriate than an approach requiring the knots to be specified in advance. Computations were carried out on a workstation equipped with an AMD Ryzen 9 5950X 16-core processor (3.40 GHz) and 64 GB of RAM, running Windows 10 Pro (version 22H2). The following software was used: the Julia programming language v.1.11.9 (open-source; https://julialang.org), including its standard packages; Splines2.jl (v.0.2.1; open-source); Survival.jl (v.0.3.0; open-source); Plots.jl (v.1.41.6; open-source); and IBM SPSS Statistics (version 27.0.1.0; IBM Corp., Armonk, NY, USA).

## 5. Conclusions

This study demonstrates that exceeding a vancomycin trough concentration threshold of 25.5 µg/mL is statistically associated with an unfavorable prognosis in ICU patients, likely reflecting both altered drug exposure and underlying illness severity rather than a direct causal effect. The use of spline Cox and segmented Cox models made it possible to characterize the nonlinear, threshold-dependent nature of this relationship and to define the concentration region beyond which a marked increase in the risk of death is observed. These findings underscore the clinical importance of vancomycin TDM as a tool for the timely detection of subtherapeutic and supratherapeutic drug concentrations. Maintaining the Ctrough within the target range may help reduce the risk of adverse outcomes in critical patients, which highlights the need for systematic monitoring of vancomycin pharmacokinetic parameters in routine clinical practice.

## Figures and Tables

**Table 1:** Anthropometric and laboratory characteristics of the patients included in the study.

Variable	Mean	95% CI (Lower)	95% CI (Upper)	Median	Min.	Max.
Age, years	57.91	55.64	60.17	59.50	20.00	92.00
Height, cm	172.13	170.92	173.34	172.00	152.00	198.00
Weight, kg	82.66	79.39	85.94	80.00	40.00	250.00
BMI, kg/m^2^	27.97	26.81	29.12	26.60	14.70	86.51
Serum creatinine, µmol/L	100.82	88.12	113.52	74.00	18.00	623.00
Follow-up time, days	28.41	25.96	30.85	24.00	3.00	88.00
Mean vancomycin conc., µg/mL	21.64	19.19	24.09	17.69	1.24	125.90
Trough (Ctrough), µg/mL	15.07	13.23	16.91	11.88	1.01	85.75
Peak (Cmax), µg/mL	29.42	25.57	33.26	23.00	1.45	231.75

**Table 2:** Distribution of patients by clinical syndrome (site of infection).

Clinical Syndrome	*n*	%
Infective endocarditis	16	8.4%
Skin and soft tissue infections	100	52.6%
Lower respiratory tract infections	79	41.6%
Thoracic/destructive lung disease	33	17.4%
Intra-abdominal infections	27	14.2%
Sepsis	72	37.9%

**Table 3:** Distribution of patients by microbiological characteristics.

Pathogen	*n*	%
*S. aureus*	83	43.7%
*S. epidermidis*	17	8.9%
*E. faecalis*	33	17.4%
*E. faecium*	20	10.6%
*P. aeruginosa*	37	19.5%
*K. pneumoniae*	78	41.1%
*A. baumannii*	52	27.4%
*P. mirabilis*	13	6.9%

**Table 4:** Distribution of patients by comorbidities.

Comorbidity	No, *n* (%)	Yes, *n* (%)
Malignant neoplasms	171 (90.0%)	19 (10.0%)
Chronic heart failure	140 (73.6%)	50 (26.4%)
Diabetes mellitus	132 (69.5%)	58 (30.5%)
Substance/alcohol abuse	176 (92.6%)	14 (7.4%)
Systemic diseases	175 (92.1%)	15 (7.9%)
Surgical interventions	92 (48.4%)	98 (51.6%)

**Table 5:** Cox regression coefficients for the final model.

Predictor	B	SE	Wald	df	*p*	Exp(B)
ln(Ctrough)	0.497	0.226	4.821	1	0.028	1.644
Age, years	0.018	0.009	4.363	1	0.037	1.019
Creatinine, µmol/L	0.004	0.001	13.101	1	<0.001	1.004

**Table 6:** Distribution of fatal outcomes by vancomycin Ctrough relative to the 25.5 µg/mL threshold.

Ctrough	Survived, *n* (%)	Died, *n* (%)	Total
<25.5 µg/mL	120 (73.6%)	43 (26.4%)	163
≥25.5 µg/mL	10 (37.0%)	17 (63.0%)	27
Total	130 (68.4%)	60 (31.6%)	190

**Table 7:** Cox regression coefficients for the model with a binary Ctrough predictor (<25.5 vs. ≥25.5 µg/mL).

Predictor	B	SE	Wald	df	*p*	Exp(B)
Ctrough ≥ 25.5 µg/mL	−0.632	0.327	3.724	1	0.054	0.532
Creatinine, µmol/L	0.004	0.001	13.835	1	<0.001	1.004
Age, years	0.022	0.009	6.531	1	0.011	1.022

**Table 8:** Mobile phase gradient throughout a chromatographic run (flow rate 1.0 mL/min).

Analysis Time, min	Volume Fraction of Eluent B, %
0.0 → 0.50	2
0.50 → 2.00	2 → 20
2.00 → 2.10	20 → 100
2.10 → 3.50	100
3.50 → 3.90	100 → 2
3.90 → 5.00	2

**Table 9:** Detection parameters for vancomycin and eremomycin in multiple reaction monitoring (MRM) mode.

Analyte	Precursor Ion, *m*/*z*	Fragment Ion, *m*/*z*	Collision Energy, V
Vancomycin	725.70	144.00	−18.0
Eremomycin (internal standard)	520.00	144.10	−12.0
	520.00	100.00	−25.0

**Table 10:** Matrix factor and extraction recovery for vancomycin and eremomycin (*n* = 8).

QC Level	Vancomycin	Eremomycin	Normalized	RSD, %
Mean Matrix Factor, *n* = 8				
Low	0.71	0.76	0.93	1.84
High	0.73	0.79	0.92	5.49
**Mean Recovery, *n* = 8**				
Low	0.93	0.96	0.97	5.76
High	0.95	1.04	0.91	2.71

**Table 11:** Within-run and between-run accuracy and precision for vancomycin.

QC Level	Within-Run (*n* = 5)		Between-Run (*n* = 15)	
	Accuracy, %	Precision, %	Accuracy, %	Precision, %
LLOQ	98.88	9.52	100.59	9.19
Low	102.08	4.31	103.76	3.94
Medium	93.78	4.29	92.12	5.02
High	88.11	3.64	89.85	3.32

**Table 12:** Stability of vancomycin under various conditions.

Stability Condition	QC Level Low (3.00 µg/mL)	QC Level High (75.00 µg/mL)
	Accuracy, %	
**Bench-top** (Room temp., 6 h)	90.89	98.55
**Post-preparation** (+4 °C, 24 h)	97.42	98.41
**Freeze–thaw** (3 cycles)	96.09	99.72
**Frozen plasma samples** (−20 °C, 7 days)	96.53	100.81
**Stock solutions** (−20 °C, 193 days)	95.64	98.76
**Working solutions** (−20 °C, 193 days)	89.82	99.53

## Data Availability

The data presented in this study are available upon request from the corresponding author. The data are not publicly available owing to privacy restrictions.

## Abbreviations

The following abbreviations are used in this manuscript:

AKI	Acute kidney injury
AUC	Area under the concentration–time curve
Cmax	Maximum concentration
Css	Steady-state concentration
Ctrough	Threshold trough concentration
GFR	Glomerular filtration rate
HPLC-MS/MS	High-performance liquid chromatography–tandem mass spectrometry
HR	Hazard ratio
ICU	Intensive care unit
IDSA	Infectious Diseases Society of America
MIC	Minimum inhibitory concentration
MRSA	Methicillin-resistant *Staphylococcus aureus*
TDM	Therapeutic drug monitoring
Vd	Volume of distribution
